# Seroprevalence of Anti-SARS-CoV-2 Antibodies in Benadir Region, Somalia

**DOI:** 10.3390/vaccines10020220

**Published:** 2022-01-30

**Authors:** Mohamed Hussein Adam, Jamal Hasan Mohamoud, Abdiaziz S. Mohamood, Ahmed A. Mohamed, Bashiru Garba, Najib Isse Dirie

**Affiliations:** 1 Department of Public Health, Faculty of Medicine and Health Sciences, SIMAD University, Mogadishu 2526, Somalia; ganowyare@simad.edu.so (M.H.A.); coofle@simad.edu.so (J.H.M.); 2 Department of Microbiology and Laboratory Science, Faculty of Medicine and Health Sciences, SIMAD University, Mogadishu 2526, Somalia; dr.abdiaziz@simad.edu.so; 3 Faculty of Medicine and Surgery, Somali National University, Mogadishu P.O. Box 15, Somalia; aadam@snu.edu.so; 4 Institute for Medical Research, SIMAD University, Mogadishu 2526, Somalia; garba.bashiru@udusok.edu.ng; 5 Department of Veterinary Public Health and Preventive Medicine, Faculty of Veterinary Medicine, Usmanu Danfodiyo University Sokoto, Sokoto 840212, Nigeria; 6 Department of Urology, Dr. Sumait Hospital, Faculty of Medicine and Health Sciences, SIMAD University, Mogadishu 2526, Somalia

**Keywords:** COVID-19, seroprevalence, anti-SARS-CoV-2 antibodies, Mogadishu, Somalia

## Abstract

Only little is known about the true extent of COVID-19 in Somalia. The study aims to assess the seroprevalence of the COVID-19 pandemics in the Benadir region using SARS-CoV-2 antibodies and estimate the number of inhabitants infected with SARS-CoV-2. Population-based cross-sectional survey was conducted to measure the seroprevalence of antibodies against SARS-CoV-2 in the Benadir region (Mogadishu city). In the study, we enrolled 2500 Mogadishu city residents aged ≥18 years who did not receive the SARS-CoV-2 vaccine. The overall seroprevalence of IgG/IgM anti-SARS-CoV-2 antibodies was 44.8%. The seropositivity in females (56.6%) was higher than in males (46.2%). The trend in seropositivity increased with age; however, the variation was only significant in the age group 38–57 with an odds ratio and *p*-value of 4.11 (1.475–11.47), *p* = 0.007. Families with >5 members (47.2%) were more likely to test positive than those with <5 members (37%). Participants who reported COVID-19 symptoms during the pandemics or who had contact with COVID-19 patients had significantly increased IgG prevalence. Participants with larger families, individuals working in the public sector, and students showed significant seropositive results. Therefore, precautionary measures should be heightened for individuals working in the public sector.

## 1. Introduction

Coronavirus 19 disease (COVID-19)—a severe, acute respiratory syndrome caused by the severe acute respiratory syndrome coronavirus 2 (SARS-CoV-2)—was first identified in Wuhan, China, in December 2019, and spread within months to most nations of the world [1,2]. By 16 August 2020, this pandemic disease was affecting people in 213 countries and territories, with about 21 million confirmed cases and around 800,000 deaths reported globally [1].

The full impact of the COVID-19 pandemic in Africa is challenging to know. Africa recorded only 2.6% (6 million) of the 233,503,524 cases reported globally by the WHO. The spread of the disease and the actual number of cases may be even higher since it is correlated with the number of tests performed, and Africa suffers from low testing capacity; therefore, the actual disease burden may be underestimated [3,4].

Somalia, a country in East Africa that is one of the poorest countries in the world and is currently recovering from a decade’s old civil war that has shattered its healthcare system, might be the least prepared country for COVID-19. Somalia reported its first confirmed case of COVID-19 on 16 March 2021 [5]. At the time of this writing, the Somali Ministry of Health reported 20,577 positive cases and a death toll of 1137 in the whole country [6]. However, this disparity is perhaps due to a lack of systematic testing of the population. It’s widely believed that the actual numbers of COVID-19 cases and deaths are much higher than the official numbers. Lack of awareness and absence of systematic testing has limited the efforts to control the spread of the disease and monitor its spread among the general population.

Moreover, people with COVID-19 have had a wide range of symptoms reported—ranging from mild symptoms to severe illness including fever or chills, cough, shortness of breath or difficulty breathing, fatigue, muscle or body aches, headache, new loss of taste or smell, sore throat, congestion or runny nose, nausea or vomiting, diarrhea [7]. Thus, untested persons go unrecognized and can expose the disease to a large portion of the population. In addition, Somalia has one of the youngest populations globally; the median age among the Somali people is 18.5 years [8]; this age is where most infected persons become asymptomatic.

In countries affected early in the pandemic, serological surveillance was used to define cumulative incidence. However, the evidence regarding COVID-19 incidence in Somalia came from the reports on newly detected infections determined by RT-PCR testing of nasopharyngeal swabs performed in selected diagnostic centers. However, no serological testing was used to monitor the epidemic on a national or regional level, and no epidemiological studies were implemented to assess the magnitude of the problem.

Our main objective was to assess the seroprevalence of the COVID-19 pandemics in Somalia using SARS-CoV-2 antibodies and estimate the number of inhabitants infected with SARS-CoV-2. The study was conducted in the Benadir region (Mogadishu City, the Capital) since it is the most populated city.

## 2. Materials and Methods

### 2.1. Participants and Study Design

A population-based cross-sectional study measuring the seroprevalence of antibodies against SARS-CoV-2 in the Benadir region (Mogadishu city) was conducted between 19 May to 29 July 2021.

The study area has an estimated 2,015,818 inhabitants, which accounts for the most populated region in Somalia. The investigators enrolled all the region districts as the primary sampling units, and each district was then divided into sub-districts and listed as the secondary sampling units. Two sub-districts from each district were randomly selected, and finally 2500 residents aged ≥18 years who did not receive the SARS-CoV-2 vaccine were recruited using a convenient sampling to participate in the study.

### 2.2. Eligibility Criteria

#### 2.2.1. Inclusion Criteria

All individuals living in Benadir region aged ≥18 years were identified for potential recruitment into the investigation; also individuals who didn’t receive the SARS-CoV-2 vaccine were recruited.

#### 2.2.2. Exclusion Criteria

Individuals who refused to give informed consent, or from whom it was not obtainable, were excluded from the study.

### 2.3. Questionnaire and Data Collection

The data were collected during the second wave of COVID-19 pandemic. Samples were collected by a three-person trained team containing a junior doctor, lab technician, and a public health final year student (one to fill the questionnaire, another to collect the biological samples for diagnostic tests, and the last one to supervise the work). All field workers were trained in protocol procedures, filling up questionnaires, and sample collection procedures under COVID-19 biosafety guidelines [9]. Consenting participants completed a questionnaire about sociodemographic, epidemiological, clinical characteristics, and laboratory data. Questionnaires were applied either in physical or digital format using an XLS form file.

### 2.4. Specimen Collection

We used IgM/IgG rapid test kit (Wondfo Biotech, Guangzhou, China), with the SARS-CoV-2 Antibody Test (Lateral Flow Method). The assay has a sensitivity of 87.12% and specificity of 99.74%, but most importantly, it targets the N-protein (nucleocapsid protein) whose IgG correlates with natural infection. To perform the assay, 20 uL of whole blood was placed on the test strip. The test strip contains a colloidal gold-labeled recombinant novel coronavirus antigen and quality control antibody colloidal gold marker, two detection lines (G and M lines) and one quality control line (C) fixed on a nitrocellulose membrane. M is fixed with monoclonal anti-human IgM antibody for detecting the novel coronavirus IgM antibody. G is fixed with monoclonal antihuman IgG antibody for detecting the novel coronavirus IgG antibody. The quality control antibody is fixed on the C line.

#### 2.4.1. Informed Consent

The purpose of the investigation was explained to all individuals identified for recruitment into the investigation. Verbal consent was obtained from all individuals willing to participate in the investigation before any procedure was performed as part of the investigation by a trained investigation team member. Each participant was informed that participation in the investigation is voluntary and that she/he is free to withdraw, without justification, from the investigation at any time without consequences and without affecting professional responsibilities.

#### 2.4.2. Risks and Benefits

The study poses minimal risk to the participants; it involves collecting a minimal amount of blood. The main benefit of the study is indirect; the data collected will help improve and guide efforts to understand the extent of COVID-19 virus infection and may prevent further transmission of the virus.

### 2.5. Data Analysis and Management Tools

The data was cleaned, coded, entered, and analyzed using statistical package for social sciences (SPSS) version 25.0 software after the questions and variables were reviewed for completeness, consistency, and accuracy. Descriptive statistics, such as frequencies and percentages, were generated and displayed in graphical and tabular formats. Furthermore, a binary logistic regression was used to investigate the association between the dependent variable “COVID-19 test result” and the respondents’ socio-demographic characteristics, history of chronic comorbidities, lifestyle, and COVID-19 preventative practice. All significance tests were two-tailed, with statistical significance set at a level of 5% alpha.

## 3. Results

### 3.1. Socio-Demographic Characteristics of COVID-19 Seroprevalence

Two thousand five hundred ninety-six individuals were eligible for the study, and 2400 (92.4%) were enrolled, whereas 196 were missing some information and excluded from the data. The number of female participants (1241) was slightly higher than the number of male participants (1115). Moreover, their ages ranged from 18 to 97, which were stratified into four age groups. Additionally, the participants were distributed across 16 districts in the Benadir area. The overall seroprevalence of IgG/IgM anti-SARS-CoV-2 antibodies in the region was 44.8%, where 1074 individuals were positive, and 1326 were negative. The seropositivity in females (56.6%) was higher compared to the males (46.2%). However, the variation was insignificant as the odds ratio, and *p* values were 0.89 (0.757–1.046), *p* = 0.156. The age group 18–37 years, had the lowest seropositivity, 40.5%, compared to the age groups of 38–57, 58–77, and 78–97, who had 56.3%, 62.7%, and 73.7% seropositivity levels, respectively. Moreover, the trend in seropositivity increased with age; however, the variation was only significant in the age group 38–57 with an odds ratio and *p*-value of 4.11 (1.475–11.47), *p* = 0.007.

The seropositivity also exhibited substantial variation according to marital status. The singles had the least seropositivity (39%), whereas the widows had the highest cases (71.0%). The married (47.9%) and the divorced (52.8%) also had higher seropositivity than the singles. Moreover, the variation in odds ratio values was significant at *p* = 0.05 in the divorced group, 2.663 (1.217–5.830), *p* = 0.014, and married group, 3.823 (1.742–8.387), *p* = 0.001.

The level of education had no statistically significant association (*p* > 0.05) with seropositivity. Notably, the primary school individuals had the highest prevalence (53.2%), whereas the high school group had the least positive cases (41.5%). Additionally, the odds ratio values ranged from 1.085 (0.798–1.476) to 1.232 (0.891–1.704) apart from the primary group at 0.767 (0.523–1.125).

Furthermore, individuals who never went to school (44.6%) had a lower proportion of seropositive individuals compared to postgraduates (46.6%) and Bachelor’s degree holders (44.3%). Therefore, the level of education had no association with seropositivity.

The seropositivity also varied with the type of occupation and family size. Primarily, the public sector (52.4%) had the highest seropositive cases compared to the students (39.5%), teachers (46.8%), self-employed (46.5%), and company workers (50.2%). Nonetheless, the variation at *p* = 0.05 was only significant in students, at 1.687 (1.066–2.670), *p* = 0.025. Moreover, the family size also influenced the seropositivity trend. Families with more than five family members (47.2%) were more likely to test positive than those with less than five family members (37%) (Table 1).

### 3.2. Habits, Comorbidities, Family History of Chronic Disease, & COVID-19 Status

The seropositivity of SARS-CoV-2 antibodies was substantially higher in smokers (65.1%) than in non-smokers (43.8%). The variation was significant at *p* = 0.05, and the odds ratio value was 2.398 (1.604–3.587), *p* = 0.0001. Similarly, the seropositivity was higher in individuals who consume Khat (58.4%) and Shisha smokers (87.0%) compared to non-users (44.3%). In comorbidities, the IgG/IgM positive cases were higher in people with diabetes (66.5%) than in the non-diabetics (43.3%).

Overall, all comorbidities influenced seropositivity as the variation was significant at *p* = 0.05 for all the conditions. Similarly, individuals with a family history of chronic diseases also exhibited a similar trend. In all chronic illnesses, the variation in seropositivity was significant at *p* = 0.05 (Table 2).

### 3.3. Clinical Features and Preventive Measures

In symptoms associated with COVID-19, fever (75.4%) exhibited a significant variation as well as coughs (74.9%) with odds ratio values of 3.464 (3.123–3.842), *p* = 0.001, and 1.279 (1.016–1609), *p* = 0.036. Similarly, the loss of smell (78.2%), loss of taste (79.9%), and stomach-upset (83.1%) also exhibited significant variation compared with data of those without the symptoms. Moreover, individuals who wear masks regularly (47%) had lower seropositivity than those who did not wear them (53%). Similarly, those who washed their hands regularly (45.8%) had slightly lower cases than those who do not wash them (43.6%), and in each of wearing mask and hand washing, the association was not statistically significant (*p* > 0.05). Furthermore, keeping a social distance (54.2%) and avoiding handshakes (48.9%) had lower seropositivity than those who do not practice these measures (51.1%), and the variation was significant at *p* = 0.05. The seropositive cases were also higher in individuals who had contact with COVID-19 patients (67.0%) than those without contact (31.0%).

The ones close to COVID-19 hospitalized patients were 69.6%, whereas those with close contact with patients who died were 64.0%. The seropositivity exhibited significant variation in individuals with close contact with COVID-19 patients (Table 3).

### 3.4. Seroprevalence in the Different Districts in Benadir

**Figure 1:**
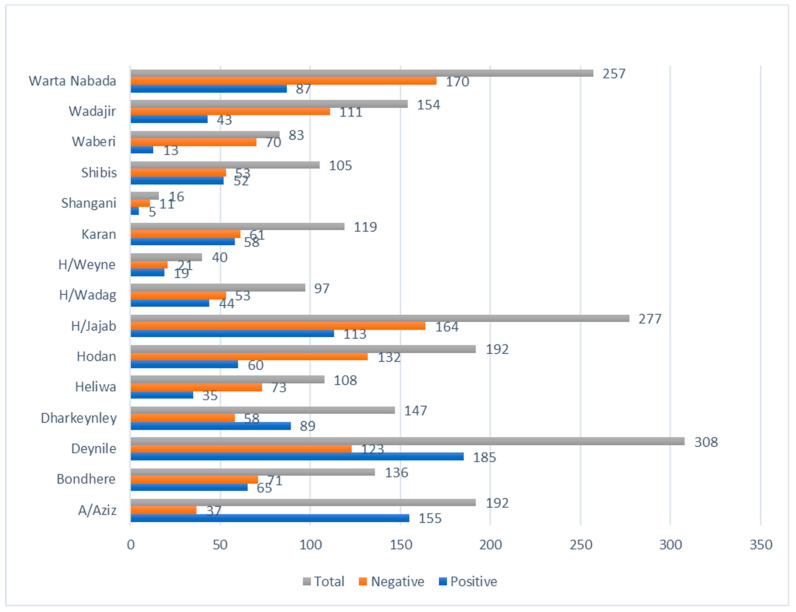
Seroprevalence of COVID-19 according to different districts of Benadir region, Somalia.

The COVID-19 antibody-positive cases in 16 districts in the Benadir region exhibited wide variation, ranging from 16 to 81%. Notably, the Abdiaziz district (81%) had the highest seropositive individuals, whereas Waberi (16%) had the least (Figure 1).

**Figure 2:**
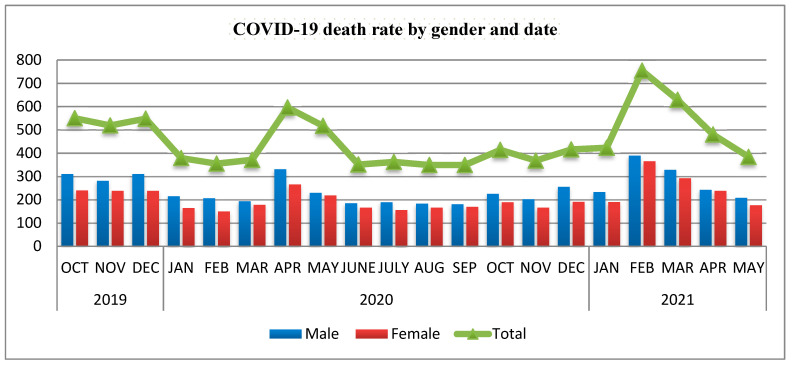
Number of people buried in Mogadishu Cemetery (Heliwa District) during COVID-19 pandemics.

Mogadishu cemetery: The data from the cemetery during different waves of pandemic is presented in Figure 2. The figure clearly shows dramatic increase of number of people buried in the cemetery during the different waves of the pandemic (First wave started 16 March 2020 and ended around late June 2020 while the second wave started late January 2021 and ended late May to early June 2021).

## 4. Discussion

The seroprevalence of IgG/IgM anti-SARS-CoV-2 antibodies in the Benadir region was 44.8%. The value is remarkably higher compared to the PCR-positive confirmed cases, as the value is slightly above 1% in Somalia [3]. The findings indicate the possibility of numerous unreported cases of COVID-19 in Somalia. It is also important to emphasize that only 5.5% of the Somali population has been vaccinated against the SARS-CoV-2 infection (WHO 2022), indicating that the circulating anti- SARS-CoV-2 antibodies are not likely due to immunity, but rather active or latent infection. The female group had a higher proportion of seropositive individuals compared to the males. However, the association of gender with seropositivity was not significant, implying it does not influence susceptibility to COVID-19. The findings are consistent with those from other regions, e.g., India, where reported seropositive cases in males (12%) were not significantly different from the females (12.8%) [10].

Similarly, Chitungo and colleagues found no association between seropositivity and gender in Poland. Additionally, the study corroborated [7], who also found no significant variation in seroprevalence of males (70%) and females (71%) in Peru. Conversely, the findings were inconsistent with (6), who suggested that the males had a higher risk of acquiring COVID-19. Nonetheless, the prevalence was consistent with the global pattern of male and female cases, showing no significant variation in the number of confirmed cases [9]. Therefore, gender has no association with seropositivity and does influence susceptibility to COVID-19 in Somalia.

The seropositive cases increased with age, as group 18–37 years had the lowest seroprevalence whereas 78–97 years had the highest prevalence. The findings suggest that older individuals are more susceptible to COVID-19 compared to younger ones. Immunosenescence (weakening of immunity with age) may be attributable to the high seropositivity in the elderly. It is characterized by a decrease in peripheral T cells in the blood, which may considerably increase susceptibility to COVID-19 [11]. Moreover, the first cases of COVID-19 in Somalia indicated similar trend where the seroprevalence in individuals aged 60 years and above (12.4, 95% CI) was higher than those who were below 20 years (5.6, 95% Cl). The Ministry of Health, Somalia observed a similar trend as the seroprevalence at 95% confidence interval increased from 1.61% in age 18–33 to 2.60% in age 65 and above. Therefore, the age of an individual influences susceptibility to COVID-19.

Marital status had an association with the seropositivity of individuals. The single individuals had a lower positive rate compared to the married, divorced, and widowed groups. Moreover, the married people had a higher association with seropositivity compared to the widowed and divorced groups. The findings imply that married individuals are at higher risk of contracting COVID-19 than the other groups. The probable reason could be that married individuals tend to cohabit with numerous relatives compared to singles.

Additionally, most males in the Somali culture are polygamous and can marry up to four wives [12]. Therefore, if a married male contracts the disease, he can transmit it to his four wives and children. Conversely, the singles are less likely to spread the disease as they tend to live alone or with fewer relatives in the house. Moreover, the divorced and widowed individuals are also expected to have children who either live with them or visit them, which increases their risk of contracting COVID-19 compared to the singles.

The groups with different levels of education had no significant variation in seroprevalence of anti-SARS-CoV-2 antibodies. Ironically, the proportion of those who tested positive in the uneducated group was comparable to those at the post-graduate level. The findings were unforeseen as the educated individuals were expected to have fewer positive cases since they are more likely to adhere to COVID-19 precautionary measures than the uneducated or those with a lower level of education. The findings suggest that the level of education does not influence susceptibility to COVID-19 in Somalia, and other factors may play a role. The findings were inconsistent with the study that reported the first case of COVID-19 in Somalia which revealed that level of education significantly influenced the seropositivity in India [10].

Similarly, the results did not corroborate with Lorent et al. who observed that individuals with a lower level of education had a higher likelihood of seropositivity [13]. The high seroprevalence in the educated individuals in the study area may be due to their occupation. Notably, the findings of this study indicated that individuals in the public sector had the highest proportion of seropositive cases. Moreover, the teachers, self-employed and those working in a company also had high seroprevalence, whereas the students had the minor positive cases. Therefore, the high seropositivity may imply that the individuals work in the public sector or other high-risk areas.

The BMI status did not influence the number of seropositive cases. The findings imply that BMI is not a risk factor for seropositivity. The results are consistent with a study in Peru, which found no significant difference in seroprevalence based on BMI status [14]. However, behavioral habits such as smoking had a significant influence on seropositivity. The findings suggest that smoking increases the likelihood of seropositivity. Notably, smoking decreases the capacity of the lungs, which heightens the risk of acquiring respiratory illnesses, including COVID-19. Primarily, SARS-CoV-2 attacks the lungs, and smoking impairs the organs, reducing one’s capacity to fight the virus [3]. Moreover, the odds ratio value was significantly high in individuals smoking shisha compared to those smoking khat. The findings imply that smoking shisha may increase one’s susceptibility to COVID-19 compared to khat. The results are inconsistent with Peckham et al., who found no correlation between smoking and COVID-19 susceptibility [15]. Additionally, we did not find a significant variation between smoking and COVID-19 prevalence as indicated by [16]. Therefore, other factors other than the COVID-19 susceptibility may have influenced the high seropositivity of smokers in Somalia.

The comorbidities such as diabetes, hypertension, asthma, and cardiovascular illnesses had a significant variation between those with the condition and the healthy individuals. Notably, the odds ratio value indicated that individuals with asthma had the highest probability of testing positive for the antibody. Additionally, individuals with other illnesses, including diabetes, hypertension, and cardiac diseases, also had an increased risk of testing positive for anti-SARS-CoV-2 antibody. The high seropositivity of individuals with asthma may be because the disease affects the lungs and is characterized by difficulty in breathing. Therefore, individuals with the diseases are likely to be more susceptible to the virus.

Moreover, the trend was similar in individuals who had a family history of the disease as they all showed significant variation compared to those who did not have a family history of the disease. The findings imply that chronic illnesses increase one’s susceptibility to COVID-19 in Somalia. The results were inconsistent with the study that reported the first COVID-19 case in Somalia, which found no significant variation between the seroprevalence of individuals with diabetes and hypertension and those who did not suffer from the illnesses [10]. The findings were inconsistent with Napoli et al., who found no association between seropositivity and comorbidities such as diabetes and hypertension [17]. The significant association between the comorbidities and COVID-19 observed in Somali may be due to lack of access to proper medication for individuals with an underlying condition, which increases their susceptibility to other illnesses such as COVID-19.

The clinical features, including fever, cough, loss of smell, and loss of taste, were all associated with seropositivity. The proportion of individuals who tested positive was substantially high in all groups (70%). Hence, the symptoms could be risk factors for COVID-19 infection in Somalia. The high association between seropositivity and the clinical features implies that the individuals may have been infected with COVID-19 when they were experiencing the symptoms. The findings are consistent with [18], who found that loss of taste and smell was associated with high seropositivity. Conversely, the same study reported that coughs and fever were not associated with seropositivity, whereas chills, fatigue, and sore throat also exhibited significant variation. Therefore, stakeholders should urge individuals experiencing the symptoms associated with COVID-19 to undertake the PCR diagnosis test for timely administration of intervention.

The routine preventive measures of COVID-19 were found to decrease seropositivity in this study. It is important to note that, although the prevalence of COVID-19 among respondents that do not frequently wear a mask and those that also don’t wash their hands frequently was higher compared to respondents that practice both of these measures, the difference observed in this study was not statistically significant. Conversely, keeping social distance and avoiding handshakes had a significant association. The results gives further credence to the already established fact that regular washing of hands and wearing masks are the hallmark of COVID-19 prevention measures. Though the association between wearing facemask and hand washing was not significant, it doesn’t conflict with the established findings, especially because the number of COVID-19 cases was higher in both groups of respondents that do not practice hand washing and wearing facemasks. The findings imply that keeping social distance and avoiding handshakes are more effective in preventing COVID-19 than wearing masks and regular washing of hands in Somalia. The probable reasons could be that individuals who wore masks were less cautious with the other measures such as keeping social distance; hence, they contracted the disease.

Additionally, the individuals washing their hands regularly were probably shaking hands with other individuals or not maintaining social distance leading to the high seroprevalence. The findings are inconsistent with other studies, as Filho et al. reported a significant association between washing hands, wearing masks, and reduction in seropositivity [19]. Nonetheless, the findings on the reduction of seroprevalence through social distancing corroborated this study. Therefore, the Somali government should emphasize keeping social distance and avoiding handshakes as additional measures to wearing masks and washing hands to curb COVID-19 prevalence.

Close contact with individuals who tested positive for COVID-19 had an association with seropositivity. Moreover, close contact with COVID-19 patients had a higher association compared to those without contact. The seroprevalence may be high since contact is one channel of COVID-19 transmission and contact with infected individuals increases one’s likelihood of contracting the illness. Moreover, keeping a social distance prevents one from contacting the infected individuals as some are asymptomatic and may be unaware of their COVID-19 positive status. Therefore, the government should sensitize citizens to self-isolate or avoid close contact with individuals who have tested positive for COVID-19 until the PCR test confirms they are negative.

The seropositive cases in 16 districts in Benadir revealed substantial variation as some districts had a high prevalence of 81%, whereas, in some regions, the prevalence was as low as 16%. The findings indicate that residents in some areas are at higher risk of contracting the disease than others. Notably, the A/Aziz district (81%) had a considerably higher seroprevalence compared to the rest. The region includes port of Mogadishu, a business hub, and has a population of 2,388,000. Therefore, the high seropositivity may be due to community transmission of the disease.

## 5. Conclusions

Despite the absence of a gold standard diagnostic test for COVID-19, serological screening has proven to be useful especially during epidemiological surveys to identify potential risk factors as observed in the present investigation. However, recent reports by Saeed and colleagues at the 2021 Annual Meeting (17–19 October 2021) of the Association for the Advancement of Blood and Biotherapeutics have argued that there is a significant limitation to the use of a single assay to estimate the serological prevalence of SARS-CoV-2 infection.

Nonetheless, in socio-demographic characteristics, the seropositivity in this study had no association with gender and level of education. The association between seropositivity and age group 38–57 years was significant, although the elderly had the highest prevalence. Therefore, the vaccination strategies in Somalia can prioritize individuals aged 38 years and above. Moreover, the public sector had the highest seropositivity in occupation, whereas the students had the least. Therefore, precautionary measures should be heightened for individuals working in the public sector.

Additionally, the larger family size was also associated with seropositivity, as was smoking and comorbidities such as asthma, cardiac diseases, hypertension, and diabetes. Hence, the individuals with these conditions can be prioritized in the administration of vaccines. Furthermore, the seropositivity was also associated with fever, coughs, stomach upset, loss of smell, and taste. Thus, individuals experiencing these symptoms in Somalia should be encouraged to undertake the PCR COVID-19 test. Moreover, the preventative measures, including wearing masks and washing hands, were not effective independently and should be combined with keeping social distance and avoiding handshakes to reduce the prevalence of COVID-19 in Somalia.

Furthermore, contact with COVID-19 patients should be avoided as it was associated with high seropositivity. Lastly, the districts with a prevalence below 40% can be targeted for vaccination. The individuals in these districts are at higher risk of COVID-19 since their seropositivity is low, implying less protection against COVID-19. Hence, the vaccination exercise should prioritize Waberi, Hodan, and Shangani districts to prevent high COVID-19 prevalence in these regions.

## Figures and Tables

**Table 1:** Socio-demographic and COVID-19 results.

Characteristics	Total (%)	Antibody Positive (IgG/IgM)	Antibody Negative (IgG/IgM)	Odds Ratio 95% CI	*p* Value
Gender					
Female	1241 (51.8)	538 (50.2)	703 (5.1)	Ref	
Male	1155 (48.2)	534 (49.8)	621 (46.9)	0.89 (0.757–1.046)	0.156
Age					
18–37	1805 (75.6)	731 (68.4)	1074 (81.5)	Ref	
38–57	444 (18.6)	250 (23.4)	194 (14.7)	4.11 (1.475–11.47)	0.007
58–77	118 (4.9)	74 (6.9)	44 (3.3)	2.17 (0.769–6.136)	0.143
78–97	19 (0.8)	14 (1.3)	5 (0.4)	1.665 (0.561–4.938)	0.358
Marital status					
Single	1005 (41.9)	392 (36.6)	613 (46.3)	Ref	
Married	1237 (51.6)	592 (55.3)	645 (48.7)	3.823 (1.742–8.387)	0.001
Divorced	123 (5.1)	65 (6.1)	58 (4.4)	2.663 (1.217–5.830)	0.014
Widowed	31 (1.3)	22 (2.1)	9 (0.7)	2.181 (.930–5.116)	0.073
Educational level					
Postgraduate	22 (9.2)	103 (9.6)	118 (8.9)	Ref	
Never went to school	628 (26.2)	280 (26.1)	348 (26.3)	1.085 (0.798–1.476)	0.604
Primary	201 (8.4)	107 (10.0)	94 (7.1)	0.767 (0.523–1.125)	0.174
Intermediate	123 (5.1)	53 (4.9)	70 (5.3)	1.153 (0.739–1.797)	0.530
High school	451 (18.8)	187 (17.5)	264 (20.0)	1.232 (0.891–1.704)	0.206
Bachelor	770 (32.2)	341 (31.8)	429 (32.4)	1.098 (0.813–1.483)	0.541
Occupational status					
Public Sector	82 (3.5)	43 (4.0)	39 (3.0)	Ref	
Student	706 (29.9)	279 (26.2)	427 (32.9)	1.687 (1.066–2.670)	0.025
Teacher	173 (7.3)	81 (7.6)	92 (7.1)	1.252 (0.740–2.120)	0.402
Self-Employed	1138 (48.2)	529 (49.8)	609 (47.0)	1.269 (0.810–1.988)	0.298
Working for a company	261 (11.1)	131 (12.3)	130 (10.0)	1.094 (0.666–1.798)	0.723
Family Size					
<5	546 (22.8)	202 (18.8)	344 (26)	Ref	
≥5	1849 (77.2)	872 (81.2)	977 (74)	1.52 (1.25–1.85)	0.001
BMI Status					
≤18.49	175 (7.6)	72 (6.9)	103 (8.1)	Ref	
18.50–24.99	1245 (53.8)	559 (53.9)	686 (53.8)	0.858 (0.622–1.183)	0.349
25–29.99	690 (29.8)	323 (31.1)	367 (28.7)	0.794 (0.567–1.11)	0.179
≥30	204 (8.8)	83 (8)	121 (9.5)	1.019 (0.676–1.537)	0.929

**Table 2:** Habits, Co-morbidities, Family history of chronic disease and COVID-19 status.

Characteristic	Total (*n* = 2388)	Antibody Positive	Antibody Negative	Odds Ratio (95% CI)	*p*-Value
		IgG/IM) (*n* = 1069)	(IgG/IGM) (*n* = 2388)		
Habits (*n*, %)					
Smoking	109 (4.6)	71 (6.6)	38 (2.9)	2.398 (1.604–3.587)	0.0001
Khat	77 (3.2)	45 (4.2)	32 (2.4)	1.767 (1.115–2.801)	0.015
Shisha	46 (1.9)	40 (3.7)	6 (0.5)	8.507 (3.593–20.142)	0.0001
Co-morbidities (*n*, %)					
Diabetes	167 (7)	111 (10.3)	56 (4.3)	2.516 (1.808–3.502)	0.001
Hypertension	172 (7.2)	109 (10.2)	62 (4.7)		
Asthma	80 (3.4)	58 (5.4)	22 (1.7)	3.364 (2.045–5.534)	0.00001
Cardiac disease	62 (2.6)	36 (3.4)	26 (2)	1.724 (1.034–2.874)	0.037
Family history/Co-morbidities (*n*, %)					
Diabetes	637 (26.6)	359 (33.5)	278 (21)	1.891 (1.574–2.271)	0.0001
Hypertension	670 (28)	363 (33.9)	307 (23.2)	1.693 (1.414–2.026)	0.01
Asthma	129 (5.4)	84 (7.8)	45 (3.4)	2.413 (1.664–3.498)	0.001
Cardiac disease	153 (6.4)	100 (9.3)	53 (4)	2.463 (1.747–3.472)	0.0001
Obesity	106 (4.4)	64 (6)	42 (3.2)	1.935 (1.300–2.881)	0.001

**Table 3:** Clinical features, preventive measures and COVID-19 status.

Characteristic	Total (*n* = 2388)	Antibody Positive (IgG/IgM) (*n* = 1069)	Antibody Negative (IgG/IgM) (*n* = 2388)	Odds Ratio 95% CI	*p*-Value
Clinical features (*n*, %)					
Fever	870 (36.3)	656 (61.1)	214 (16.1)	3.464 (3.123–3.842)	0.001
Cough	757 (31.7)	567 (52.8)	190 (14.3)	1.279 (1.016–1.609)	0.036
Loss of smell	747 (31.1)	584 (54.4)	163 (12.3)	1.660 (1.257–1.192)	0.0001
Loss of taste	618 (25.8)	494 (46)	124 (9.4)	1.613 (1.205–2.159)	0.001
Stomach-upset	166 (6.9)	138 (12.8)	28 (2.1)	1.392 (1.005–1.927)	0.046
Preventive Measures of COVID-19 (*n*, %)					
Wearing Mask	1007 (42.3)	473 (4.3)	534 (40.6)	1.161 (0.986–1.367)	0.073
Wash Hands Regularly	1253 (52.2)	574 (53.7)	679 (51.4)	1.095 (0.931–1.278)	0.272
Keep Distance From Others	633 (26.6)	312 (29.3)	321 (24.4)	1.279 (1.066–1.535)	0.008
Avoid Handshake	810 (33.9)	396 (37)	414 (31.4)	1.284 (1.083–1.522)	0.004
Close Contact with					
COVID-19 Patient	796 (33.4)	549 (51.5)	247 (18.8)	4.592 (3.823–5.516	0.0001
Relative, colleagues, friends had COVID-19	913 (38.2)	612 (57.2)	301 (22.8)	4.528 (3.795–5.403)	0.001
Hospitalized for COVID19	700 (29.3)	487 (45.6)	213 (16.2)	4.348 (3.597–5.257)	0.0001
Died patient from COVID-19	606 (25.4)	388 (36.3)	218 (16.5)	2.871 (2.371–3.476)	0.0001

## Data Availability

The data presented in this study are available in the article.
